# CF Patients’ Airway Epithelium and Sex Contribute to Biosynthesis Defects of Pro-Resolving Lipids

**DOI:** 10.3389/fimmu.2022.915261

**Published:** 2022-06-16

**Authors:** Mickael Shum, Charlie M. London, Maelle Briottet, Khadeeja Adam Sy, Vincent Baillif, Reginald Philippe, Abdolhossein Zare, Sadegh Ghorbani-Dalini, Natacha Remus, Agathe Tarze, Virginie Escabasse, Ralph Epaud, Marc Dubourdeau, Valerie Urbach

**Affiliations:** ^1^ University Paris Est Créteil, Institut National de la Santé Et de la Recherche Médicale (INSERM) U955, Institut Mondor de Recherche Biomédicale (IMRB), Créteil, France; ^2^ Ambiotis, Toulouse, France; ^3^ Institut National de la Santé Et de la Recherche Médicale (INSERM) U1151 – Institut Necker Enfants Malades (INEM), Paris, France; ^4^ Centre Hospitalier Intercommunal de Créteil (CHIC), Créteil, France

**Keywords:** specialized pro-resolving mediators, cystic fibrosis, gender specificity, lipidomics, resolution of inflammation

## Abstract

Specialized pro-resolving lipid mediators (SPMs) as lipoxins (LX), resolvins (Rv), protectins (PD) and maresins (MaR) promote the resolution of inflammation. We and others previously reported reduced levels of LXA4 in bronchoalveolar lavages from cystic fibrosis (CF) patients. Here, we investigated the role of CF airway epithelium in SPMs biosynthesis, and we evaluated its sex specificity. Human nasal epithelial cells (hNEC) were obtained from women and men with or without CF. Lipids were quantified by mass spectrometry in the culture medium of hNEC grown at air-liquid interface and the expression level and localization of the main enzymes of SPMs biosynthesis were assessed. The 5-HETE, LXA4, LXB4, RvD2, RvD5, PD1 and RvE3 levels were significantly lower in samples derived from CF patients compared with non-CF subjects. Within CF samples, the 12-HETE, 15-HETE, RvD3, RvD4, 17-HODHE and PD1 were significantly lower in samples derived from females. While the mean expression levels of 15-LO, 5-LO and 12-LO do not significantly differ either between CF and non-CF or between female and male samples, the SPMs content correlates with the level of expression of several enzymes involved in SPMs metabolism. In addition, the 5-LO localization significantly differed from cytoplasmic in non-CF to nucleic (or nuclear envelope) in CF hNEC. Our studies provided evidence for lower abilities of airway epithelial cells derived from CF patients and more markedly, females to produce SPMs. These data are consistent with a contribution of CF airway epithelium in the abnormal resolution of inflammation and with worse pulmonary outcomes in women.

## Introduction

Chronic inflammation can stem from a failure to eliminate a pathogen, a dysfunctional immune system or from failure to resolve inflammation after an initial injury (1, 2). In cystic fibrosis (CF), the most common life-threatening human genetic disease, abnormalities in the resolution process of inflammation involving specialized pro-resolving lipid mediators (SPMs) have been proposed to contribute to the sustained airway inflammation (3–8).

The SPMs constitute a subcategory of oxylipins, i.e. oxidized fatty acids that differ from others by their role as cell signaling molecules. The resolution of acute inflammation previously believed to be a passive process by dilution and/or clearance of pro-inflammatory mediators and neutrophils apoptosis, is currently described as an active phase orchestrated by SPMs with many studies bringing evidence of their pro-resolving actions in various diseases (9–16). They are the counterpart of originally identified pro-inflammatory lipid mediators such as prostaglandins (PG) and leukotrienes (LT). The main families of SPMs that have been identified to date are lipoxins (LX), resolvins (Rv), protectins (PD) and maresins (MaR). They limit inflammation by inhibiting the synthesis and function of pro-inflammatory cytokines as well as leukocyte chemotaxis and migration. SPMs enhance innate microbial killing and clearance by increasing leukocytes’ phagocytosis capacity. They stimulate macrophages’ efferocytosis and promote tissue regeneration (17). SPM biosynthesis results from the cooperation between different cell types selectively expressing various enzymes such as lipoxygenases, and exchanging intermediates to achieve the final metabolites (18–20). The substrates for these enzymes are arachidonic acid (AA), an ω**_6_** polyunsaturated fatty acid (PUFA); docosahexaenoic acid (DHA) and eicosapentaenoic acid (EPA), both ω**_3_**PUFAs. SPMs were found in various locations including plasma or serum as well as sputum, saliva, tears, breast milk, urine, synovial and cerebrospinal fluids, adipose tissue, skeletal muscle, hippocampus, skin, placenta, lymphoid tissues, and atherosclerotic plaques. Differences in SPM concentrations have been reported between health and disease with SPM concentrations found reduced in some affections and higher in others. Most of the studies were performed on mixed groups of males and females (21). The few studies that explored gender differences reported controversial data (22–26).

CF is a recessive monogenic disease due to mutations in the *Cystic Fibrosis Transmembrane conductance Regulator* (*CFTR*) gene, encoding a chloride channel (27). The airway disease is the main cause of morbidity and mortality in CF patients. Among more than 2000 *CFTR* mutations, two thirds of patients have at least one copy of the F508del mutation (28). The commonly admitted model of CF airway pathogenesis is that the ion transport abnormalities lead to defective mucociliary clearance and favor chronic pathogen colonization associated with neutrophilic inflammation, generating bronchiectasis and progressive lung function decline (29). However, in CF airways, the inflammatory response to bacteria is exaggerated and studies suggested that inflammation can occur without primo-infection (30–33). Furthermore, a worse pulmonary outcome is reported in CF female patients, although the involved mechanisms remaining unclear. Some studies started to shed light on mechanisms behind the sustained inflammatory airway disease notably with the studies of SPMs levels in CF. The concentrations of the SPM LXA4 in sputum were found significantly suppressed in patients with CF compared to patients with other inflammatory lung conditions (3). An imbalance between LXA4 and leukotriene B4 (LTB4), a pro-inflammatory lipid was found in the bronchoalveolar lavage (BAL) fluids from children with CF (4). RvD1 has also been proposed as a marker of CF airway disease (7, 34). However, to our knowledge, the biosynthesis of other SPMs and an eventual gender specificity has not been explored in CF.

In the present study, we investigated the role of CF airway epithelial cells in SPMs biosynthesis, using primary cultures of nasal samples from non-CF and CF subjects and the CFBE41o- cell line. Moreover, we explored sex differences in the ability of CF airway epithelial cells to produce SPMs.

## Materials and Methods

### Study Population

Nasal polyp or mucosa samples from 19 non-CF and 18 CF donors of the *Centre Hospitalier Intercommunal de Créteil* (CHIC, France) were excised after informed consent. 22 women and 15 men were included. Most of the patients were homozygous for F508del with 3 patients carrying other mutations. The non-CF donors were older than the CF patients (**Table 1**). All experiments were performed in accordance with the Declaration of Helsinki and the Huriet-Serusclat and Jardet law on human research ethics. The study of these human samples was approved for the by the *Comité de Protection des Personnes Ile de France* (ID-RCB: 2011-A00118-33).

**Table 1:** Donor’s characteristics.

	Total			Lipidomics		
CFTR genotype	Number	Age	Gender	Number	Age	Gender
WT	19	48	13F/6M	15	44	10F/5M
F508del homozygous	15	23	8F/7M	13	23	7F/6M
F508del heterozygous	3	23	1F/2M	3	23	1F/2M
**Total**	37	–	22F/15M	31	–	18F/13M

Number, median age in years (yrs) and ratio of females (f) over males (M) recruited within each CFTR genotype group. Among the 37 recruited subjects (left), the samples from 31 of them were used for lipidomics analysis (right).

### Cell Culture

Isolated cells from biopsies were grown in PneumaCult™ medium (StemCell) at air liquid interface (ALI) to reconstitute a pseudo stratified epithelium. After being surgically excised, biopsies were kept in a transport medium containing equal parts of Dulbecco’s Modified Eagle Medium with 0.45% glucose (DMEM, ThermoFisher Scientific) and Ham’s F-12 nutrient medium (ThermoFisher Scientific) supplemented with antimicrobial agents: penicillin-streptomycin (200 U/mL), 0.01% gentamycin and 0.01‰ amphotericin B (PSGA, all from ThermoFisher Scientific). The delay between surgery and dissociation did not exceed 4 hours. The samples were then washed with a phosphate-buffered saline (PBS) solution containing PSGA and dithiothreitol (DTT, Euromedex) to remove mucus and blood, including immune cells. Samples were then put in the transport medium supplemented with 0.01% pronase (Sigma-Aldrich) overnight at 4°C. The pronase solution was then replaced by fetal cow serum (FCS) medium composed of equal parts DMEM and Ham’s F-12 medium, 5% FCS and PSGA. After agitating and filtering out the remaining tissue, the medium was centrifuged, and the supernatant removed. A trypsin-ethylenediaminetetraacetic acid (EDTA) 0.05% Phenol Red solution (ThermoFisher Scientific) was added to the cells for 3 minutes then deactivated with an equal volume of FCS medium. The remaining solution was centrifuged again, and the supernatant removed. The cell pellet was suspended in a complete expansion medium containing PneumaCult™-Ex Basal Medium (StemCell), Pneumacult-Ex supplement, PSGA and hydrocortisone (final concentration 9.6 µg/100 mL, StemCell), seeded at a density of 2.5 x 10^5^ in T25 flasks and kept at 37°C and 5% CO_2_. The flasks and all subsequent culture surfaces were previously coated with collagen IV from human placenta (Sigma-Aldrich). Once cell confluency reached 80%, cells were isolated using 0.025% trypsin, and seeded on permeable filters (12-well Transwell™ plates) at a cell density of 2 x10^5^ cells/cm² in Pneumacult™-Ex Medium was added. Once cell confluency was reached, the apical medium was delicately removed, and the basal chamber medium was switched to an air-liquid interface (ALI) maintenance medium composed of Pneumacult™-ALI Basal Medium, Pneumacult™-ALI supplement, hydrocortisone, and heparin (StemCell). After a minimum of 4 weeks of ALI culture, we could observe a pseudo stratified epithelium with basal cells, columnar ciliated cells and goblet cells secreting mucus (**Supplementary Figure 1**).

Immortalized CFBE41o^−^ bronchial epithelial cells, with expression of wild-type (WT) and F508del-CFTR were provided by Dr Gruenert (35). Cells were cultured on fibronectin coated permeable filters in MEM supplemented with 10% FBS, 1% of Tazocilline and 300 µg/mL of Hygromycin B and incubated at 37°C in a 5% CO_2_ containing atmosphere, as previously described (36).

### Lipidomic Analysis

The lipid mediators were quantified in the basolateral medium from cell cultures obtained from 15 non-CF subjects, 13 F508del homozygous and 3 CF patients carrying other mutations. The medium of the basal chamber was collected after 4 to 5 weeks of cell culture in ALI and 3 days after the culture medium was renewed. PUFA metabolites were also analyzed in the culture medium without being exposed to cells but kept in the same condition (37°C and 5% CO_2_) for 3 days (**Supplementary Figure 2**). All samples were fast frozen and stored at -80°C for a maximum of 3 months before lipidomic analysis to prevent alteration of lipid content.

Twenty-four oxylipids were quantified by Ambiotis^©^, using very sensitive and selective targeted liquid chromatography-tandem mass spectrometry (LC-MS/MS) metabololipidomics. This includes the AA metabolites, LXA4, LXB4, LTB4, PGE2, TXB2, 6-K-PGF1A, 15-hydroxyeicosatetraenoic acid (HETE), 5-HETE, 12-HETE, the EPA metabolites, 18-hydroxyeicosapentaenoic acid (HEPE), RvE1, RvE2, RvE3, and the DHA metabolites, RvD1, RvD2, RvD3, RvD4, RvD5, MaR1, MaR2, PD1, PDX, 17-hydroxy docosahexaenoic acid (HDOHE) and 14-HDOHE. The extraction and analysis were performed as previously described (26). Samples were taken to solid-phase extraction and lipid mediators were eluted in HCOOMe. After solvent evaporation, samples were dissolved in MeOH and injected into an Agilent 1290 Infinity high-performance liquid chromatography (HPLC) system equipped with a Kinetex Biphenyl column (2.1 mm, 50 mm, 1.8 μm) (Phenomenex). The lipids were eluted with a binary gradient of water/formic acid 0.1% and acetonitrile/formic acid 0.1% and taken to MS/MS analysis on a triple quadrupole Agilent 6490 instrument. The identification of the lipid mediators in the samples was based on matching of their retention time with the retention time of pure authentic standards injected in the same conditions. Calibration curves were obtained using the same authentic standards, and quantification was carried out based on peak areas from multiple reaction monitoring (MRM) transitions. Peak detection, integration, and quantitative analysis were done using Mass Hunter Quantitative Analysis Software (Agilent Technologies). Quantification results were expressed in pg/mL.

### Western Blotting

Enzyme expression levels were assessed using western blotting on protein extracts derived from ALI primary cultures from 11 non-CF and 13 CF as well as the CFBE14o^−^ bronchial epithelial cells expressing WT and F508del-CFTR. Cells were washed and harvested by scratching in cold RIPA buffer (ThermoFisher Scientific) complemented with 10% protease inhibitor cocktail (P8340, Sigma-Aldrich). Protein samples were then collected by a 10 min centrifugation at 4°C, 14 000g, and the pellet was discarded. Protein concentration was measured using Bradford assay. 20 µg of protein extracts were mixed with Laemmli buffer and ran on a 10% polyacrylamide gel. The proteins were transferred to a 0.2 µm Polyvinylidene Fluoride (PVDF) membrane in a liquid transfer buffer (Tris, Glycine and Methanol buffer) at 4°C for 16 to 18 hours. The membrane was then saturated in blocking buffer (5% fat dry milk dissolved in a Tris-buffered saline and 0.1% Tween-20 solution (TBST) and incubated overnight at 4°C with primary antibodies. After the washes, membranes were incubated for 1h at RT with secondary antibodies coupled with HRP and washed again. Staining was revealed using the Supersignal™ West Pico PLUS Chemiluminescent Substrate kit (ThermoFisher Scientific). Images were analysed and bands densitometry measured using the ImageJ software. The following antibodies dilutions were used: anti-5-LO 1:200 (Cayman, No. 160402), anti-15-LO1 1:200 (SantaCruz, sc-133085), anti-15-LO2 1:200 (SantaCruz, sc-271290), anti-12-LO 1:500 (Abnova, H00000242-A01), and anti-β actin 1:5000 (Sigma-Aldrich, A5316). Secondary antibodies were diluted at 1:10000. All antibodies were diluted in the blocking buffer. The protein ladder used was Spectra™ Multicolor High Range (ThermoFisher Scientific).

### Immunofluorescence

The immunofluorescence analyses were performed on airway epithelial cells cultured either on glass coverslip and referred as 2D cultures, or on permeable filters and referred as 3D cultures. For 2D cultures, cells were plated on glass coverslips for 24h prior to fixation. Fixation was performed by incubating cells with ice-cold acetone for 7 minutes and was followed by several washes. For 3D cultures, cells grown for 4-5 weeks on permeable filters were washed with PBS, and fixation was performed by incubating with Carnoy solution (60% ethanol, 30% chloroform and 10% glacial acetic acid). After overnight incubation, the filter of each insert was carefully removed using scalpels and tweezers, then placed in cassettes for embedding. Paraffin embedding and 4 µm sectioning were performed by the IMRB imaging platform. After deparaffinization, non-specific sites were blocked using a blocking buffer of 3% BSA diluted in PBST, for 1h. Staining was performed by incubating primary antibodies overnight at 4°C in a humid chamber. Following antibodies dilutions were used: anti-5-LO 1:50 (Cayman, No. 160402), anti-15-LO1 1:100 (antibodiesOnline, ABIN2455524), anti-15-LO2 1:100 (SantaCruz, sc-271290), anti-Calreticulin 1:200 (Enzo, ADI-SPA-600-F) and anti-cPLA2 1:100 (SantaCruz, sc-438). All antibodies were diluted in blocking buffer. After PBS washes, the slides were incubated with fluorescent secondary antibodies diluted in PBS-T completed with 5% goat serum for 1h at RT, in obscurity. The following antibodies were used in accordance with primary antibody species: goat anti-mouse conjugated with AF488, goat anti-rabbit conjugated with AF488 and goat anti-rabbit conjugated with AF594 (Abcam, A11001, A11034 and A11012, respectively), all diluted to 1:1000. After PBS washes, coverslips were mounted on glass slides using a mounting medium containing DAPI (ProLong™ Gold Antifade reagent with DAPI, Sigma-Aldrich). Images were acquired using a confocal microscope (LSM 900 Airyscan 2) and analyzed using the ImageJ software.

### ELISA for IL-8 Quantification

IL-8 levels in basolateral cell culture medium were determined by ELISA, following instructions from Human IL-8/CXCL8 ELISA Kit (RAB0319-1KT, Sigma-Aldrich). The culture medium samples were collected in the same fashion as those used for lipidomic analysis. Briefly: the cell culture medium diluted to the hundredth was loaded onto a human IL-8 antibody-coated 96 wells plate. A biotinylated IL-8 antibody was left to incubate in the wells, then an HRP-Streptavidin solution was used to target the biotin. Optical density reading at 450 nm was performed with a TECAN Infinite 200 Pro microplate reader.

### Statistical Analysis

Results are expressed as means ± SEM and compared with the nonparametric Mann-Whitney two-sided test. The Spearman and Pearson tests were used for correlation analyses. *P* values of <0.05 were considered statistically significant. All statistical analyses were performed using GraphPad Prism 9 software (GraphPad Software, San Diego, CA).

## Results

### Lipid Mediators’ Biosynthesis by Airway Epithelial Cells

**Figure 1:**
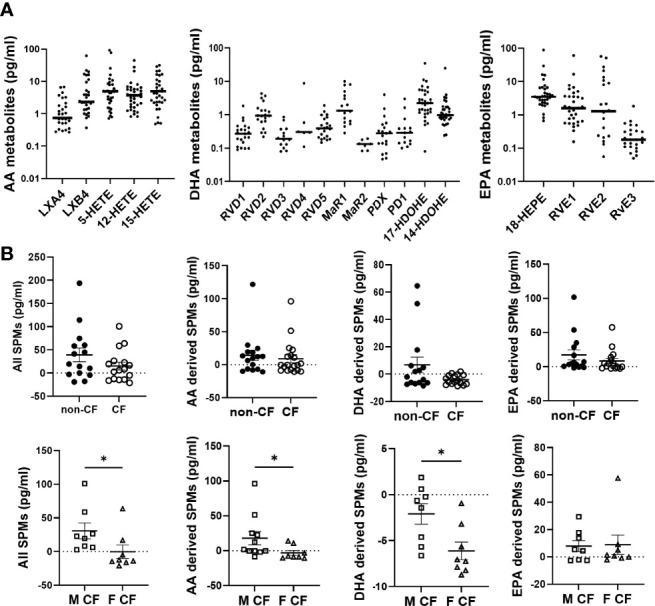
Lipid mediators detected in CF and non-CF hNEC culture medium. **(A)** Raw values of arachidonic acid (AA), docosahexaenoic acid (DHA) and eicosapentaenoic acid (EPA) metabolites measured in the basolateral culture medium of CF and non-CF hNEC. **(B)** Comparison of the cumulative levels of all lipid mediators, of AA metabolites, of DHA metabolites and of EPA metabolites between hNEC culture medium derived from non-CF and CF subjects and from males (M) and females (F) with CF (after subtraction of values obtained in cell free medium). (Mann-Whitney test. *p<0.05).

Most of the lipids were detected at the pg/mL range in the basolateral medium of hNEC primary cultures (**Figure 1A**). Since PUFA metabolites were detected in the culture medium without being exposed to cells (**Supplementary Figure 2**), the amounts measured in cell-free medium were subtracted from the values obtained in the cell culture medium. This rendered negative values at times, suggesting that cells’ consumption of metabolites was higher than their production (**Figure 1B**). The cumulative levels of SPMs or of each PUFA pro-resolving metabolites analyzed (AA, DHA, EPA) showed a trend towards decrease in CF compared with non-CF hNEC samples (**Figure 1B**, top). Within CF samples, the cumulative quantities of all SPMs and of AA and DHA metabolites were found significantly lower in females than in males (**Figure 1B**, bottom). These metabolites were further analyzed, separately.

### Arachidonic Acid Metabolites

**Figure 2:**
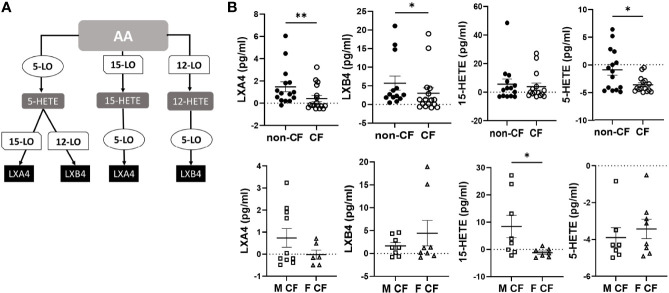
Arachidonic acid (AA) derived SPMs biosynthesis. **(A)** Schematic illustration of the lipoxin A4 (LXA4) and lipoxin B4 (LXB4) biosynthesis’ pathway showing the enzymes involved, 5-lipoxygenase (LO), 15-LO and 12-LO as well as their intermediates substrates, 5- hydroxyeicosapentaenoic acid (HETE), 15-HETE and 12-HETE. **(B)** Comparison of the mean levels of each AA metabolite (LXA4, LXB4, 5-HETE and 15-HETE) between samples derived from non-CF and CF subjects (top) and from male and female CF patients (bottom). (Mann-Whitney test. *p<0.05), **p<0.01).

The AA can be oxygenated by the 5-LO, the 15-LO and the 12-LO and reduced to 5-HETE, 15-HETE and 12-HETE, which are further metabolized by the15-LO, 12-LO and 5-LO, to produce LXA4 and LXB4 (**Figure 2A**). The amounts of LXB4 from all sample groups (CF and non-CF) were higher than the amount of LXA4 produced. The 15-HETE and 12-HETE were significantly produced compared with 5-HETE which was more consumed than produced (**Figure 2B** and **Table 2**). The mean LXA4, LXB4 and 5-HETE levels were significantly lower in CF hNEC medium than in non-CF samples, while 12-HETE and 15-HETE levels did not differ significantly (**Figure 2B** and **Table 2**). Within CF samples, LXA4, 12-HETE and 15-HETE levels were lower in females than in males (**Figure 2B** and **Table 2**).

**Table 2:** Arachidonic acid (AA) metabolites biosynthesis.

	LXA4	LXB4	15-HETE	12-HETE	5-HETE
**non-CF**	**1,474 ± 0.45**	**5.72 ± 1.89**	5.61 ± 3.59	4.40 ± 4.62	**-0.93 ± 0.97**
**CF**	**0,4135 ± 0.27**	**3.04 ± 1.44**	3.92 ± 2.89	7.13 ± 6.98	**-3.70 ± 0.42**
***non-CF vs CF (p)***	***0.01***	***0.04***	*NS*	*NS*	***0.03***
**F non-CF**	**1.43 ± 0.57**	4.46 ± 1.88	**1.68 ± 1.95**	-0.05 ± 2.06	-1.82 ± 1.18
**M non-CF**	1.21 ± 0.73	4.79 ± 3.31	**15.44 ± 11.07**	13.33 ± 13.24	**0.85 ± 1.50**
**F CF**	**-0.02 ± 0.20**	4.44 ± 2.78	**-1.20 ± 0.52**	-2.94 ± 0.34	-3.43 ± 0.52
**M CF**	0.73 ± 0.0.42	1.65 ± 0.74	**8.40 ± 4.10**	17.21 ± 13.42	**-3.89 ± 0.52**
***F CF vs M CF (p)***	*NS*	*NS*	***0.01***	*0.08*	*NS*
***F non-CF vs M non-CF (p)***	*NS*	*NS*	***0.01***	*0.07*	*0.09*
***F non-CF vs F CF (p)***	***0.01***	*NS*	*NS*	*NS*	*NS*
***M non-CF vs M CF (p)***	*NS*	*NS*	*NS*	*NS*	***0.01***

Mean levels of each AA metabolite (LXA4, LXB4, 5-HETE, 12-HETE, 15-HETE) in non-CF and CF samples and in the subgroups of female and male subjects. Bold values are related to significant differences. NS, not significant.

### Docosahexaenoic Acid Metabolites

**Figure 3:**
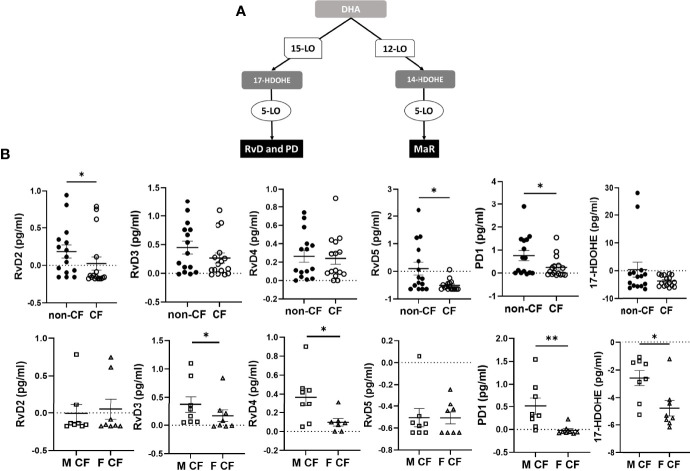
Docosahexaenoic acid (DHA) metabolites biosynthesis. **(A)** Schematic illustration of the D-series of resolvins (RvD), protectins (PD) and maresins (MaR) biosynthesis’ pathway showing the enzymes involved, 5-LO, 15-LO and 12-LO as well as their intermediates substrates 17-HDOHEand 14-HDOHE. **(B)** Comparison of the mean levels of each DHA metabolite (RvD2, rvD3, RvD4, RvD5, PD1 and 17-HDOHE) between samples derived from non-CF and CF subjects (top) and from male and female CF patients (bottom). (Mann-Whitney test. *p<0.05), **p<0.01).

The DHA can be metabolized by the 12-LO and 15-LO, respectively, to 14-hydroxy docosahexaenoic acid (HDOHE) and 17-HDOHE, two intermediates giving rise to the D-series Rv, PD and MaR (**Figure 3A**). The RvD2, RvD5, PD1 were significantly lower in CF samples than non-CF. Within CF samples, RvD3, RvD4, PD1 and 17-HODHE were significantly lower in females than in males, while these lipid amounts were not significantly different between male and female samples from the non-CF population (**Figure 3B** and **Table 3**).

**Table 3:** Docosahexaenoic acid (DHA) metabolites biosynthesis.

		RvD1	RvD2	RvD3	RvD4	RvD5
**non-CF**		0.13 ± 0.04	**0.18 ± 0.09**	0.44 ± 0.11	0.26 ± 0.06	**0.09 ± 0.22**
**CF**		0.13 ± 0.05	**0.02 ± 0.08**	0.26 ± 0.08	0.24 ± 0.05	**-0.50 ± 0.04**
***non-CF vs CF (p)***		*NS*	***0.02***	*NS*	*NS*	***0.03***
**F non-CF**		0.09 ± 0.04	**0.24 ± 0.11**	0.46 ± 0.15	**0.31 ± 0.08**	0.14 ± 0.33
**M non-CF**		*0.22 ± 0.08*	***0.06 ± 0.11***	*0.41 ± 0.15*	*0.15 ± 0.07*	*-0.01 ± 0.20*
**F CF**		0.18 ± 0.10	**0.05 ± 0.13**	**0.16 ± 0.10**	**0.09 ± 0.04**	-0.50 ± 0.05
**M CF**		0.08 ± 0.04	-0.004 ± 0.11	**-0.36 ± 0.14**	**0.36 ± 0.09**	-0.50 ± 0.05
***F CF vs M CF(p)***		*NS*	*NS*	***0.05***	***0.05***	*NS*
***F non-CF vs M non-CF (p)***		*NS*	***0.05***	*NS*	*NS*	*NS*
***F non-CF vs F CF (p)***		*NS*	***0.01***	*NS*	***0.04***	*NS*
***M non-CF vs M CF (p)***		*0.07*	*NS*	*NS*	*0.07*	*0.09*
* *		**MaR1**	**MaR2**	**PD1**	**14-HDOHE**	**17-HDOHE**
**Non-CF**		-0.79 ± 0.07	-0.02 ± 0.02	**0.76 ± 0.22**	5.24 ± 3.01	0.37 ± 2.70
**CF**		-0.83 ± 0.08	-0.03 ± 0.0.01	**0.24 ± 0.11**	0.01 ± 0.29	-3.69 ± 0.47
***non-CF vs CF***		*NS*	*NS*	***0.04***	*NS*	*NS*
**F non-CF**		-0.83 ± 0.06	-0.04 ± 0.01	**0.49 ± 0.20**	**1.48 ± 1.43**	-3.70 ± 0.68
**M non-CF**		*-0.70 ± 0.21*	*-0.04 ± 0.01*	*1.30 ± 0.47*	*12.77 ± 7.00*	*8.53 ± 6.30*
**F CF**		-0.76 ± 0.05	-0.03 ± 0.01	**-0.01 ± 0.04**	**-0.57 ± 0.27**	**-4.79 ± 0.56**
**M CF**		-0.90 ± 0.04	-0.05 ± 0.01	**0.5 ± 0.18**	0.60 ± 0.44	**-2.63 ± 0.44**
***F CF vs M CF***		*NS*	*NS*	***0.001***	*0.07*	***0.003***
***F non-CF vs M non-CF***		*NS*	*NS*	*NS*	*NS*	*NS*
***F non-CF vs F CF***		*NS*	*NS*	***0.004***	***0.03***	*NS*
***M non-CF vs M CF***		*NS*	*NS*	*NS*	*NS*	*NS*

Mean levels of each DHA metabolite (RvD1-5, MaR1-2, PD1 14-HODHE, 17-HODHE) in non-CF and CF samples and in the subgroups of female and male subjects. Bold values are related to significant differences. NS, not significant.

### Eicosapentaenoic Acid Metabolism

**Figure 4:**
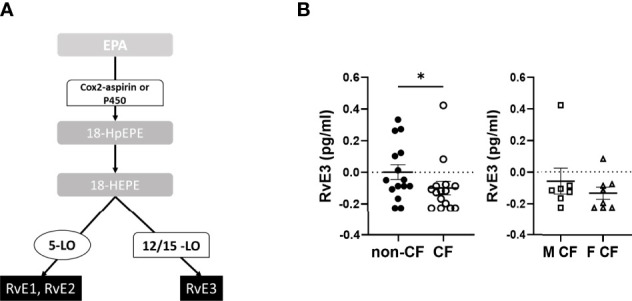
Eicosapentaenoic acid metabolism (EPA) metabolites biosynthesis. **(A)** Schematic illustration of the E-series of resolvins (RvE) biosynthesis’ pathway showing the enzymes involved, 5-LO, 15-LO, 12-LO and their intermediates substrates, the 18-hydroxyeicosapentaenoic acid (18HEPE). **(B)** Comparison of the mean levels of the EPA metabolite RvE3 between samples derived from non-CF and CF subjects (left) and from male and female CF patients (right). (Mann-Whitney test. *p<0.05).

The EPA gives rise to 18-hydroxyeicosapentaenoic acid (HEPE), the precursor of the E-series Rv. The initial EPA metabolite is not produced by a LOX activity but either by an aspirin acetylated cyclooxygenase (COX) 2 or cytochrome P450 (**Figure 4A**). The E-series Rv appeared at low concentrations in the hNEC medium, but the obtained values were above the limit of quantification. The RvE3 concentrations were significantly lower in CF compared with non-CF hNEC samples but did not show any differences between males and females with CF (**Figure 4B** and **Table 4**).

**Table 4:** Eicosapentaenoic (EPA) metabolites biosynthesis.

	RvE1	RvE2	RvE3	18-HEPE
**Non-CF**	3.14 ± 0.55	6.98 ± 3.45	**0.00 ± 0.04**	6.18 ± 5.50
**CF**	2.78 ± 0.52	6.79 ± 3.65	**-0.09 ± 0.04**	-0.94 ± 0.63
***non-CF vs CF***	*NS*	*NS*	**0.04**	*NS*
**F non-CF**	2.92 ± 0.72	8.68 ± 5.40	-0.02 ± 0.06	1.38 ± 2.72
**M non-CF**	*3.49 ± 0.65*	*4.15 ± 2.23*	*0.05 ± 0.06*	*17.18 ± 16.93*
**F CF**	1.28 ± 0.55	8.68 ± 7.031	-0.13 ± 0.04	-0.83 ± 0.32
**M CF**	4.10 ± 1.90	4.90 ± 2.63	-0.06 ± 0.08	-1.06 ± 1.27
***F CF vs M CF***	*NS*	*NS*	*NS*	*NS*
***F non-CF vs M non-CF***	*NS*	*NS*	*NS*	*NS*
***F non-CF vs F CF***	*NS*	*NS*	*NS*	*NS*
***M non-CF vs M CF***	*NS*	*NS*	*0.05*	*0.09*

Mean levels of each DHA metabolite (RvE1, RvE2, RvE3 and 18-HEPE) in non-CF and CF samples and in the subgroups of female and male subjects. Bold values are related to significant differences. NS, not significant.

### Interleukin-8 Secretion Levels

**Figure 5:**
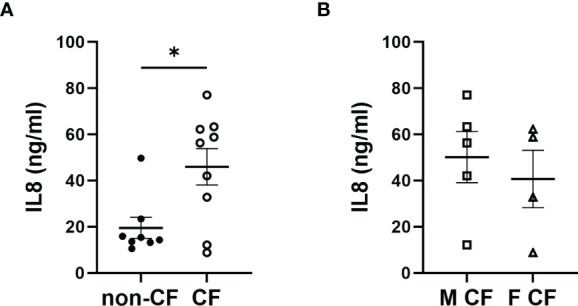
Interleukin 8 (IL8) secretion. **(A)** Comparison of IL8 secretion in hNEC cell culture medium between samples derived from non-CF and CF subjects and **(B)** from females and males CF patients. (Mann-Whitney test. *p < 0.05).

We measured the pro-inflammatory interleukin-8 (IL-8) levels in the basolateral hNEC culture medium from CF and non-CF cells. IL-8 levels were significantly higher in medium from CF samples, with no significant differences between males and females CF patients (**Figure 5**).

## Enzymes Expression Levels

**Figure 6:**
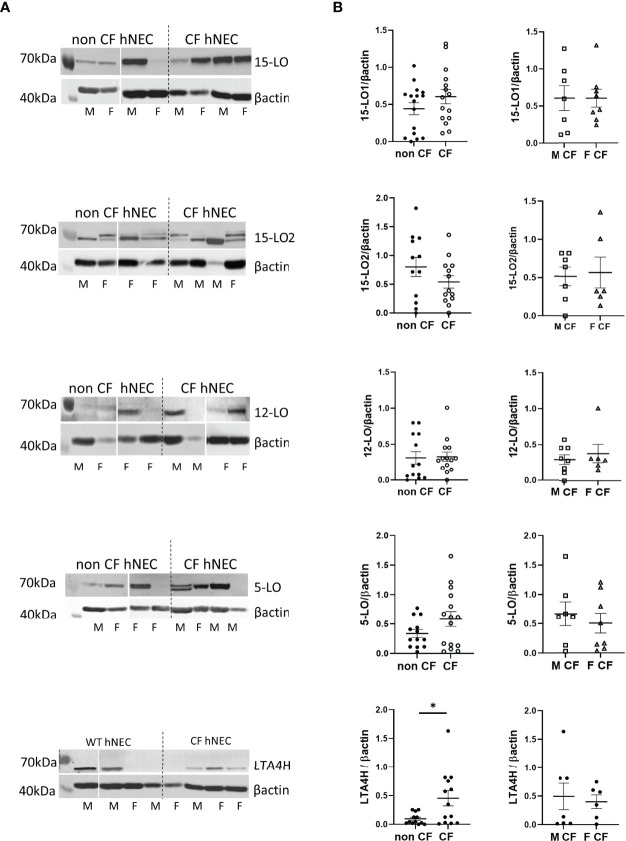
Expression levels of the 15-LO1, 15-LO2, 12-LO, 5-LO and LTA4H in hNEC. **(A)** Typical western-blotting results from protein extract of non-CF and CF female (F) and male (M) hNEC. **(B)** Comparison of the mean enzymes expression levels between non-CF and CF hNEC (left) and between CF male and female hNEC (right). *p < 0.05.

**Figure 7:**
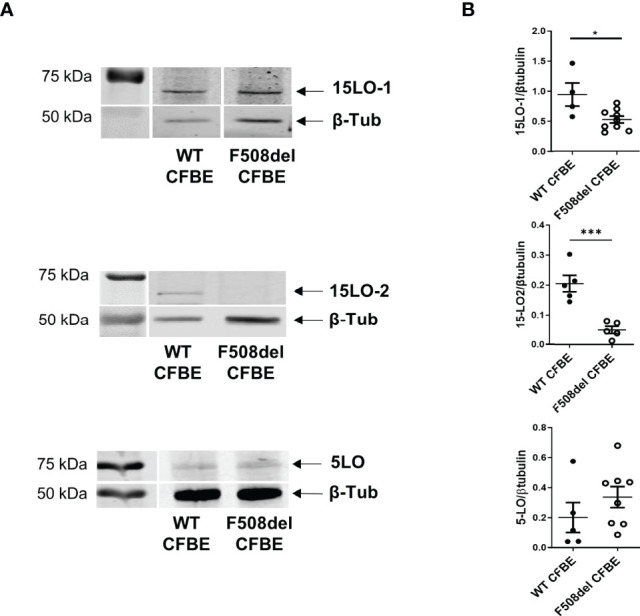
Expression level of 15-LO1, 15-LO2 and 5-LO in CFBE41o- cell line expressing WT CFTR and F508del CFTR. **(A)** Typical western-blotting results. **(B)** Comparison of the mean enzymes expression levels between both cell lines. *p < 0.05 and ***p < 0.001.

We assessed enzymes expression level on protein extracts of hNEC derived from 12 CF patients and 13 non-CF subjects. As shown in **Figures 3A**, **4A**, **5A**, the 3 PUFAs can be metabolized by LOs cooperation. In addition, the AA metabolite product from 5-LO activity (5-HETE) can also give rise to pro-inflammatory lipids– leukotrienes, through the activity of the leukotriene A4 hydrolase (LTA4H). Therefore, we investigated the expression of the two isoforms of 15-LO (15-LO1 and 15-LO2), the 12-LO, the 5-LO and the LTA4H. All these enzymes were detected in our samples. The mean expression levels of LOs were not significantly different between protein extracts from non-CF and CF hNEC primary cultures. However, the mean LTA4H concentration, was significantly higher in protein samples extracted from CF compared with non-CF hNEC. Within CF samples, the enzymes’ mean expression levels were not significantly different between hNEC cultures from males and females (**Figure 6**). In the CFBE41o- cell lines, the expression levels of 15-LO1 and 15-LO2 were significantly lower in cells expressing F508del CFTR, while 5-LO expression did not differ (**Figure 7**).

**Figure 8:**
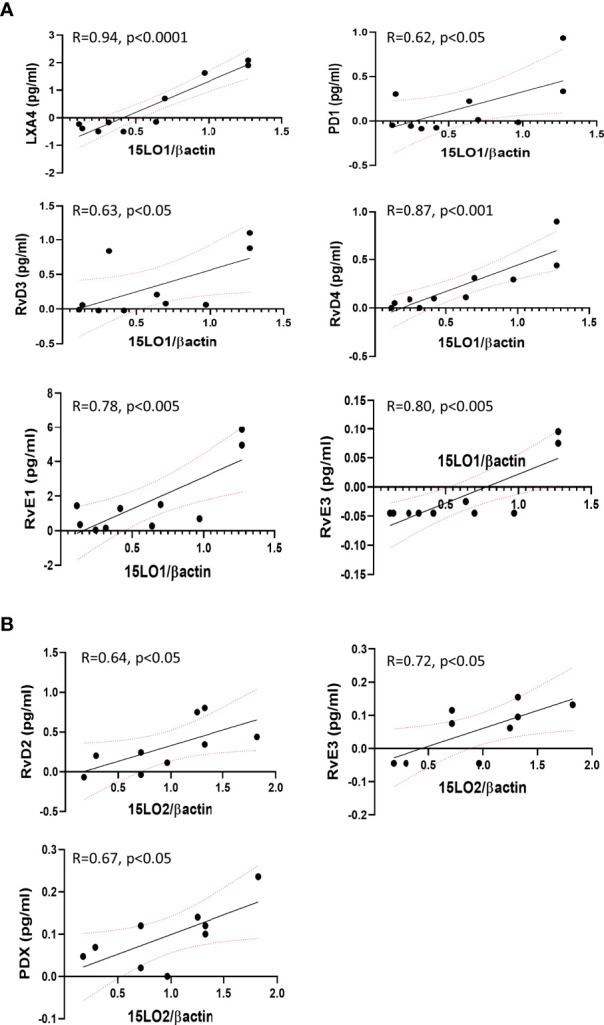
Selected significant Pearson’s correlations between enzymes expression levels and SPMs biosynthesis by the same hNEC sample. **(A)** Positive correlations between 15-LO1 and SPMs in CF hNEC. **(B)** Positive correlations between 15-LO2 and SPMs in non-CF hNEC.

Correlation analyses revealed that the level of expression of several enzymes involved in SPMs metabolism correlates with the content of some SPMs or intermediates in the same hNEC culture. We found significant correlations between 15-LO2 expression and RvD2, PDX, and RvE3 in non-CF samples. In contrast, 15-LO1 had strong and significant positive correlations with LXA4, RvD3, RvD4, RvE1, RvE3 and PD1 in CF hNEC. We found correlations between 12-LO and SPMs in non-CF and CF samples, but not as strong as 15-LO. The expression levels of 5-LO were positively correlated with PDX in non-CF and with LXB4 and RvE3 in CF hNEC. Finally, when analyzing the overall samples, we found lower but significant negative correlation between LTA4H enzyme expression level and LXB4 and RvD5, RvE3 and PD1 (**Figure 8** and **Supplementary Table 1**).

### Enzyme Localization

**Figure 9:**
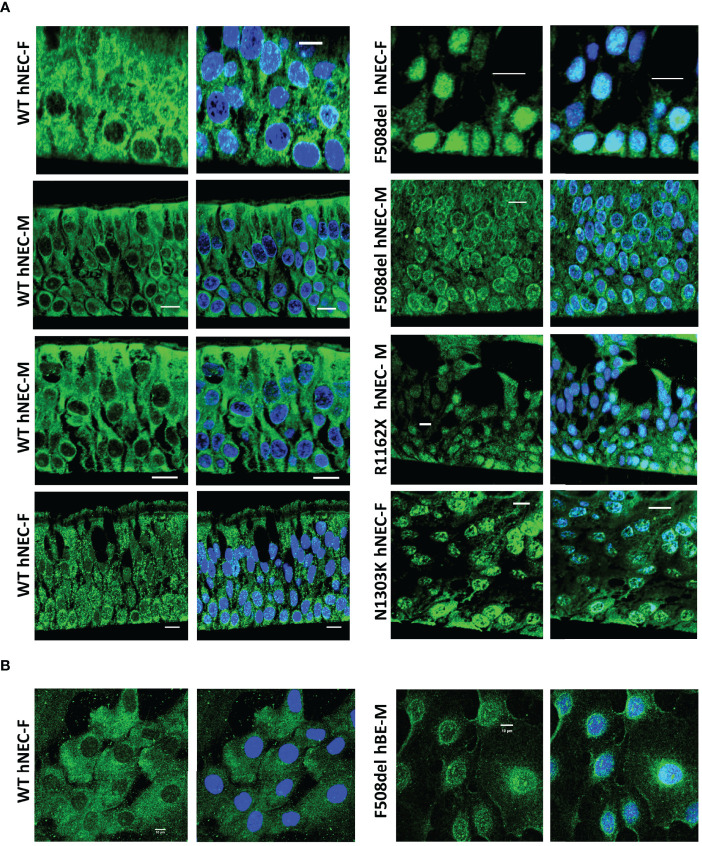
5-LO localization in hNEC. Typical 5-LO (green) immuno-fluorescence confocal images of airway (nasal: hNEC and bronchial: hBE) epithelial cells primary cultures derived from 9 different subjects with different CFTR mutations. The non-CF (WT, left) and CF (right) female (F) and male (M) airway primary cultures were **(A)** grown at ALI on permeable filters, embedded in paraffin and sliced or **(B)** grown on glass coverslips. Nucleus were stained in DAPI (blue). Bar=10µm.

**Figure 10:**
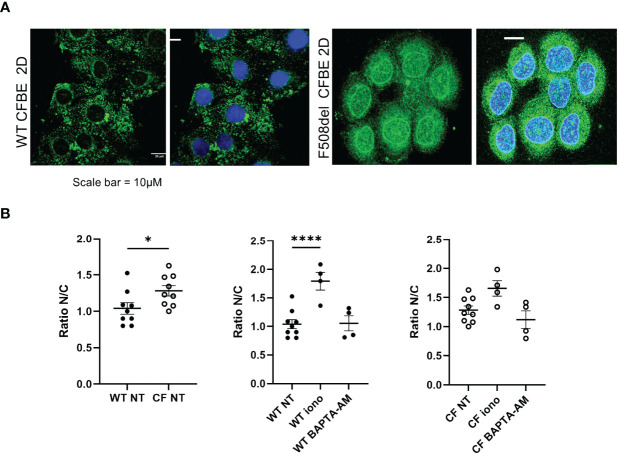
Localization of the 5-LO in the in CFBE41o- cell line. **(A)** Intracellular localization of the 5-LO in CFBE41o- cell line expressing WT CFTR and F508del CFTR grown on glass coverslips. **(B)** Effect of ionomycin and BAPTA-AM on the 5-LO localization evaluated by the quantification of the nucleus/cytoplasmic ratio of 5-LO staining. *p < 0.05 and ****p < 0.0001.

Since 5-LO localization might confer a change in its enzymatic function, we explored its localization using confocal microscopy techniques. Slides of reconstituted epithelium or isolated cells in primary cultures were immuno-stained with 5-LO antibody. The 5-LO localization changed significantly between CF and non-CF samples. Non-CF hNEC showed a 5-LO cytoplasmic localization (**Figure 9**, left) but CF primary cultures (F508del, N1303K or R1162X mutations) showed nuclear staining, in fully differentiated cells grown at ALI (**Figure 9A**) as well as in undifferentiated cells (**Figure 9B**). In some CF primary cultures, the 5-LO staining appeared stronger at the nuclear envelope. The 5-LO nuclear exclusion was not gender specific and cultures derived from males (M) and females (F) provided similar patterns (**Figure 9**). The change in 5-LO localization was also observed in the CFBE41o- cell lines. The nucleus and cytoplasm fluorescence intensity ratio showed a significant increase in F508del CFBE41o- cells compared with cells expressing WT CFTR (**Figure 10**).

Confocal microscopy analysis of 15-LO1 and 15-LO2 failed to show any difference between cells expressing WT or mutated CFTR (**Supplementary Figure 3**).

## Discussion

With this study, using LC-MS/MS and pure authentic standards which are required for a sensitive and selective oxylipin analysis, we report, for the first time, the lipidomic analyses of the SPMs biosynthesized by differentiated nasal epithelial cells (hNEC) cultured from CF and non-CF male and female donors. Our study provides evidence, for reduced SPM biosynthesis abilities of airway epithelial cells derived from CF patients and even more so in female patients.

### SPMs’ Biosynthesis by Airway Epithelial Cells

First, we demonstrate that differentiated airway epithelium derived from CF and non-CF male and female donors possess the necessary enzymes to produce a wide spectrum of SPMs, without being exposed to immune cells that also possess the machinery to produce them. Indeed, we report the expression of the related enzymes as well as their activity with the SPMs released by hNEC found in the concentration range of human plasma (21, 37). The ability of airway epithelial cells to contribute to SPMs production is of particular interest since airway inflammation mostly targets epithelial cells. Thus, the local balance between pro-inflammatory or pro-resolving mediators which results from paracrine and autocrine eicosanoids production (by immune cells and epithelial cells) will determine the fate of this tissue and its protective and barrier functions.

Although studies of SPMs biosynthetic pathways have been mainly focused on leukocytes (37), consistent with our data, the 12-LO, 15-LO and 5-LO expression has been previously reported in bronchial and nasal epithelial cells (38–40). While, the two intermediates 15-HETE and 12-HETE resulting from 15-LO and 12-LO activity respectively, were significantly more produced than consumed (positive values), the 5-HETE resulting from 5-LO activity was more consumed than produced (negative values) (**Table 2**). This suggests that LXA4 and LXB4 biosynthesis by airway epithelial cells mainly occurs *via* the initial oxygenation of AA by 12-LO or 15-LO followed by a further conversion of intermediate products by 5-LO. This pathway has been referred to as the 12/15-LO: 5-LO pathway in opposition to the 5-LO: 12/15-LO pathway in which 5-LO activity occurs first (**Figure 2A**) (37).

In our study, the concentrations of the pro-inflammatory eicosanoid LTB4 often appeared below the detection limits and thus out of the concentration range we previously observed in the BAL of human subjects (**Supplementary Figure 3**) (4). Since LTA4 (the substrate of LTA4H to produce LTB4) results from an initial AA conversion by 5-LO, the low levels of LTB4 are consistent with the low 5-LO activity of airway epithelial cells compared to their 15-LO and 12-LO activities. Indeed, the absence of leukocytes’ 5-LO activity in our cellular model reduced the balance between 5-LO and 15/12-LO activity and resulted in AA mostly used for LXA4 or LXB4 biosynthesis rather than LTB4. Other pro-inflammatory mediators TXB4 and PGE2 were also produced by hNEC at detectable levels (**Supplementary Figure 3**).

As reported by others, DHA and EPA metabolites appeared at lower concentrations than AA metabolites suggesting that these two ω**_3_**PUFAs were not as readily available as AA in cell membranes (41, 42). However, in our study and as previously reported the cell-free culture medium contained detectable amounts of LO substrates (PUFAs and intermediate metabolites). This most probably explain why although low, the DHA and EPA derived metabolites were found above detection limit. Although we did not measure all the intermediate products of DHA and EPA (5-HEPE, 15-HEPE, 7-HODHE and 4-HODHE), the D-series Rv, MaR and PD biosynthesis most likely result from the 12/15-LO: 5-LO pathway.

### Abnormal SPM Production by CF Airway Epithelial Cells

Furthermore, our study provides evidence for the impact of *CFTR* mutation on SPMs biosynthesis by airway epithelial cells. Indeed, LXA4, LXB4, RvD2, RvD5, PD1 and RvE3 released by CF hNEC appeared significantly reduced compared with non-CF hNEC. These novel data are consistent with other reports showing lower LXA4 content in the BAL of CF patients, although these previous studies were based on enzyme-linked immunosorbent assays (ELISAs) further demonstrated as less reliable methods than MS (3, 4, 7). The reduced DHA metabolites (cumulate levels, and RvD2, RvD5 and PD1) in our CF samples is consistent with Veltman *et al*, recent study (40). This team also reported increased cumulative AA metabolites in CF but did not distinguish between SPMs and pro-inflammatory eicosanoids. In our study, the overall amounts of SPM showed a trend toward decrease in CF samples (**Figure 1B**), while the pro-inflammatory mediators (LTB4, PGE2, TXB2 and 6-K-PGF1A) had the opposite trend (increase in CF) (**Supplementary Figure 3**). In addition, differences between our two studies could be also explained by the high variability of lipid detection from one patient’s sample to another and the low quantity of donors they had (3 homozygous F508del patients and 2 other mutations, while we had 13 homozygous F508del and 3 others) (40).

The levels of the intermediate metabolites 12-HETE, 15-HETE, 17-HDOHE and 14-HDOHE which result from 15-LO, 12-LO activities did not change significantly between CF and non-CF samples. This was consistent with our protein analysis showing that 15-LO1, 15-LO2 and 12-LO mean expression levels did not change significantly between both groups. Previous studies have shown contradictory results. One of them reported no difference for 15-LO expression but an increased 12-LO expression in CF nasal mucosa (16). A proteomic analysis showed an increase expression of 15-LO in nasal polyps from CF patients, however, this study was performed on a small number of subjects (4 CF and 4 non-CF) and had not been confirmed by western blotting (43). We previously reported a reduced level of 15-LO2 isoform expression in CF BAL fluids, but this was strongly correlated to the macrophages count (4). Of note, while we failed to observe any correlation between 15-LO1 expression levels and SPMs concentration in non-CF samples, we found positive correlations between 15-LO1 and several SPMs in CF hNEC. In contrast, we found positive correlations between the 15-LO2 expression levels and SPMs in non-CF samples while these correlations disappeared in CF ones (**Figure 8** and **Supplementary Table**). This might reflect the change in 15-LO1/15-LO2 ratio between non-CF and CF samples, with the 15-LO1 being more highly expressed in CF and the 15-LO2 more highly expressed in non-CF samples.

On the other hand, we showed that 5-HETE resulting from AA by 5-LO was significantly lower in CF samples. Indeed, the 5-HETE was 4 times more consumed in CF than in non-CF samples. This suggests that the reduced LXA4, LXB4, RvD2, RvD5, PD1 and RvE3 levels in CF samples were mainly due to a reduced 5-LO activity (either to produce 5-HETE from AA or to metabolize 12-HETE, 15-HETE, 17-HDOHE and 14-HDOHE) and/or an increased consumption of 5-HETE rather than a change in 12-LO or 15-LO activities. The change in 5-HETE levels were not related to a change in 5-LO expression levels since we failed to show any significant difference between non-CF and CF primary cultures, as previously reported (16). However, the higher expression of LTA4H in CF samples is consistent with an increase consumption of 5-HETE in CF samples.

Furthermore, the reduced biosynthesis of 5-HETE or its increased consumption could result from a change in 5-LO activity which has been previously described as related to its location. Indeed, the 5-LO appeared diffused in the cytoplasm of airway epithelial cells expressing WT-CFTR and was localized in the nucleus or at the nuclear envelope in cells with any *CFTR* mutation (F508del, R1162X or N1303K), regardless of the cell differentiation level. The changes in 5-LO localization have been known for a while. The 5-LO was initially described in the nuclear envelope of activated leukocytes (44). Other studies demonstrated that soluble 5-LO were predominantly in the cytoplasm of neutrophils and eosinophils, or predominantly intranuclear in alveolar macrophages (45–47). In addition, the formation of 5-LO-derived products has been described as related to the calcium-dependent translocation of the enzyme to nuclear membranes where it interacts with the 5-LO associated protein (FLAP). Elevation of intracellular calcium concentration has been used to trigger lipoxin (48, 49) as well as D-series (50) and E-series (51) Rv formation by neutrophils and macrophages. However, the balance between pro-inflammatory and pro-resolving AA-derived mediators in leukocytes is thought to be achieved through intracellular localization of 5-LO. Nuclear 5-LO favors the biosynthesis of proinflammatory LTB_4_, whereas cytoplasmic 5-LO favors the biosynthesis of pro-resolving LXA_4_. During inflammation, 5-LO is phosphorylated and translocated to the nuclear membrane, to metabolize AA to 5-HETE for its conversion to LTB4 on one hand and its extranuclear export for lipoxin generation on the other hand (52, 53). In macrophages, a 5-LO shift from the nucleus to the cytosol is induced by RvD1and stimulated LXA4 production (52). Consistent with these previous studies, we found that increase in intracellular calcium, enhanced the 5-LO nuclear localization and BAPTA-AM (an intracellular calcium chelator) decreased it (**Figure 10**) (52). As described in macrophages, the nuclear 5-LO observed in CF airway epithelial cells is consistent with lower levels of 5-HETE, LXA4, LXB4, RvD5 and RvE1 released in the culture medium of these cells.

Additionally and consistent with already reported intrinsic abnormalities of the inflammatory process within CF airway epithelial cells already reported, we observed significantly higher IL-8 levels in culture medium of CF hNEC primary culture compared with non-CF (30, 54).

One limitation of our study was the younger CF population compared to non-CF donors. However, previous studies have shown reduced levels of SPMs in blood, cells and tissues from elders compared with youngers that could predispose to age-related diseases (21). Therefore, in our study the lipidomic profile differences between CF and non-CF samples might have been underestimated. Other SPMs than the ones we highlighted, might be abnormally produced by airway epithelial cells from CF (young patients) when compared with samples from subjects of the same class of age.

### Sex Impact on SPM Biosynthesis in CF

Finally, we explored whether sex could play a role in SPM biosynthesis by airway epithelial cells. Sex specificities of SPM biosynthesis remains controverted. On one hand, a meta-analysis initially reported that females had higher blood plasma concentration of the AA and DHA metabolites 18-HEPE, 14-HODHE and 17-HODHE, but these differences disappeared in the derived SPMs levels: RvE1, RvE2, RvE3, RvD1, RvD2, PD1 or MaR1 (22, 23). Skin blisters induced by cantharidin contained metabolites from AA, EPA and DHA which were not individually significantly different in females, but the cumulative RvD levels were significantly higher in females than in males (25, 55). Other studies have shown lower levels of SPMs, plasma RvE2, RvD1 and AT-RvD1 levels in females compared to males with no difference in plasma RvD2 between sexes (21, 24, 25). Analyses of human tears also revealed sex differences as SPMs were absent in female donors (26). In our study, 15-HETE and RvD2 were found to be significantly lower in female than in male samples derived from the non-CF population (**Tables 2**, **3**).

In CF, epidemiological studies have shown a sex-based disparity in pulmonary outcomes, where females experience higher rates of exacerbation, earlier and higher lung bacterial colonization and a shortened life expectancy than males. Multiple factors including anatomical differences, socio-environmental factors, medication adherence, physical activity level and sex hormones have been suggested, but mechanisms remain to be elucidated (56–58). In our gender specific subgroup analysis, we found that the three SPMs, RvD3, RvD4 and PD1 and the intermediary metabolites 15-HETE, 12-HETE, and 17-HODHE were lower in samples from CF women than in CF men. In contrast, in the non-CF population, we found no sex differences for RvD3 and PD1. However, RvD4 showed an opposite difference, with increased levels of RvD4 in non-CF females compared with non-CF males. Therefore, this reduced ability of female CF airway epithelial cells to produce SPMs might contribute to the worse pulmonary outcomes in CF women compared with men.

Our data suggests that the differences in SPM biosynthesis abilities between CF and non-CF, and between CF female and CF male epithelial cells were not related to the same cellular mechanisms. The reduced SPMs biosynthesis by CF airway epithelial cells was consistent with the 5-LO nuclear localization and a change in this enzyme’s activity. In contrast, the mechanism involved in the lower ability of airway epithelial cells from females to produce SPMs could be related to a reduced 15-LO and 12-LO activities since the intermediate metabolites 15 and 12-HETE are reduced in samples from females with CF compared with males with CF. Therefore, CF females would cumulate alteration of 5-LO and 15/12-LO activities.

### Consequences of SPMs Abnormalities in CF Airway

SPMs exert protective biological activities to oppose excessive inflammation and tissue damage as demonstrated in several chronic inflammatory diseases including CF airway disease. Yang et al. reported a broad range of both pro-inflammatory and resolving lipid mediators in sputum from CF patients. While the associations of pro-inflammatory mediators with compromised lung function were not robust, these authors revealed that cumulated amounts of SPMs had a stronger association with lung function (8). Similarly, Eickmeier et al. reported RvD1 as a marker of the severity of the CF airway disease (7). During chronic *P. aeruginosa* infection in mice, LXA_4_ stable analog reduces PMN recruitment and bacterial burden in short-term *P. aeruginosa* murine models of lung infection (3). In airway epithelia exposed to *P. aeruginosa in vitro*, LXA_4_ protected from cell injury, strengthened tight junction integrity, and reduced IL-8 production (59, 60). LXA4 and RvD1 also target epithelial cells to restore the airway surface hydration (ASL) that is important for mucociliary clearance. Other SPMs from the D-series resolving, PD1 and LXB4 have also been shown to confine excessive inflammation and boost host defense against pathogens (5, 16, 61–69). Therefore, although it remains to be established *in vivo*, the reduced levels of the novel SPMs we report in the present study may contribute to the progression and worsening of CF airway disease.

## Conclusion

Taken together, our results demonstrate that airway epithelial cells from CF patients showed a significant alteration of SPMs biosynthesis ability with significantly reduced LXA4, LXB4, RvD2, RvD5, PD1 and RvE3 levels. These data are consistent with an altered resolution phase in the airway of patient with CF, contributing to chronic and excessive inflammation. Furthermore, the reduced ability of airway epithelial cells from CF women to produce RvD3, RvD4 and PD1 could produce an additive effect contributing to the worse respiratory outcomes in women with CF.

## Data Availability Statement

The original contributions presented in the study are included in the article/**Supplementary Material**. Further inquiries can be directed to the corresponding author.

## Ethics Statement

The studies involving human participants were reviewed and approved by Comité de Protection des Personnes Ile de France. Written informed consent to participate in this study was provided by the participants’ legal guardian/next of kin.

## Author Contributions

MS, CL, MB, RP, AT, NR, VE, RE, MD, and VU contributed to the conception and the design of the study. MS, CL, MB, VB, RP, AZ, and SG-D performed experiments. VU performed the statistical analysis and wrote the first draft of the manuscript. MS, CL, MB, RP, and KS wrote sections of the manuscript. All authors contributed to manuscript revision, read, and approved the submitted version.

## Funding

This work was funded by the *Institut National de la Santé Et de la Recherche Médicale* and the parents and patients association *Vaincre la Mucoviscidose* (#FR20180502278, #FR20190502472, #FR20200502701).

## Conflict of Interest

Authors VB and MD were employed by Ambiotis.

The remaining authors declare that the research was conducted in the absence of any commercial or financial relationships that could be construed as a potential conflict of interest.

## Publisher’s Note

All claims expressed in this article are solely those of the authors and do not necessarily represent those of their affiliated organizations, or those of the publisher, the editors and the reviewers. Any product that may be evaluated in this article, or claim that may be made by its manufacturer, is not guaranteed or endorsed by the publisher.
