# Supplementary material for: CF Patients’ Airway Epithelium and Sex Contribute to Biosynthesis Defects of Pro-Resolving Lipids
Source: Front Immunol. 2022 Jun 16;13:915261. doi: 10.3389/fimmu.2022.915261 (PMC9244846; doi:10.3389/fimmu.2022.915261)
Supplement: Supplementary file 1 [file DataSheet_1.docx]

Supplementary Material

# Supplementary Figures


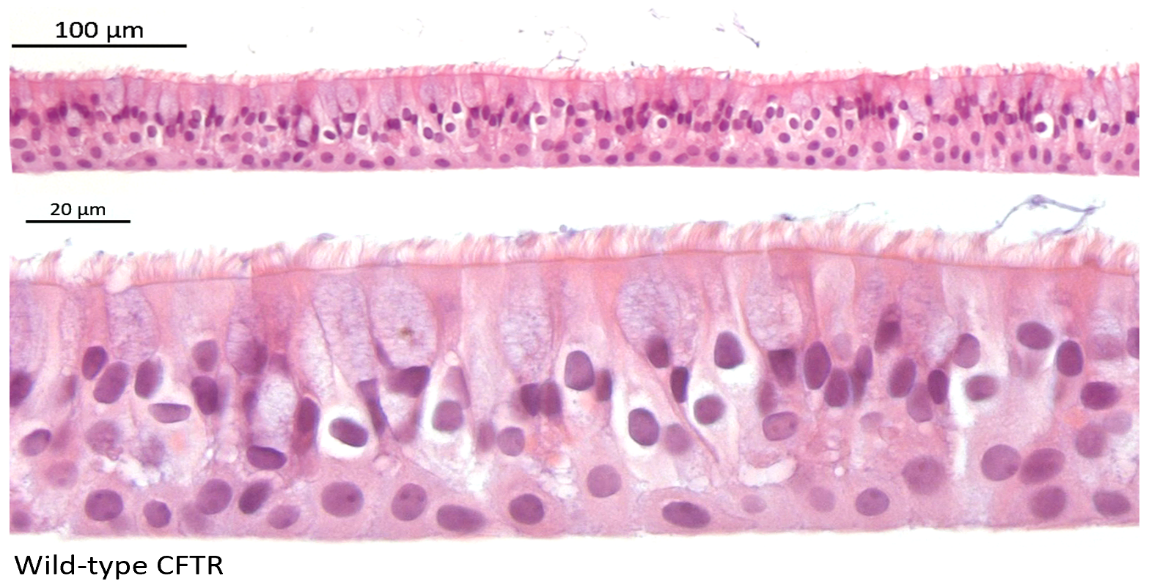


**Supplementary Figure 1.** Typical Hematoxylin and Eosin staining of a slide (4µm) of hNEC epithelium primary culture on a permeable filter at air liquid interface. The staining reveals a pseudostratified structure with cilia at the apical surface as well as the presence of goblet cells.

| **lipids** | **pg/mL** |
| --- | --- |
| LXA4 | 0,496 |
| LXB4 | 0,882 |
| 5-HETE | 5,627 |
| 12-HETE | 4,717 |
| 15-HETE | 3,571 |
| RVD1 | 0,113 |
| RVD2 | 0,176 |
| RVD3 | 0,021 |
| RVD4 | - |
| RVD5 | 0,641 |
| MaR1 | 1,001 |
| MaR2 | 0,072 |
| PDX | - |
| PD1 | 0,087 |
| 17-HDOHE | 6,711 |
| 14-HDOHE | 1,555 |
| 18-HEPE | 4,158 |
| 18R-HEPE | 1,334 |
| 18S-HEPE | 2,824 |
| RVE1 | 0,296 |
| RVE2 | *-* |
| RvE3 | 0,227 |

**Supplemental figure 2.** PneumacultTM-ALI Basal Medium, supplemented with hydrocortisone, and heparin (StemCell). The medium was kept in the incubator at 37°C and 5% CO2 for 3 days, the same conditions as the medium contained in the basolateral compartment of the cell culture inserts.


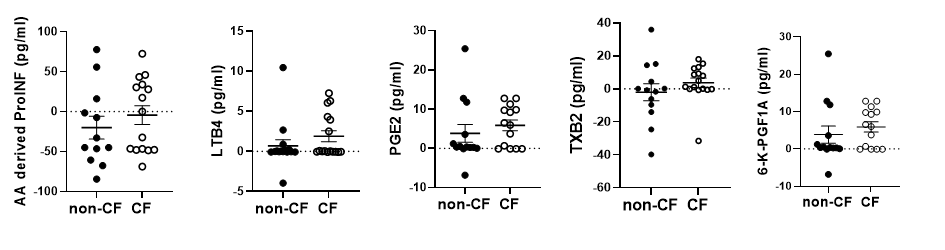


**Supplemental figure 3.** Arachidonic acid (AA) derived pro-inflammatory lipid mediators’ biosynthesis. Comparison of the cumulative levels of AA derived pro-inflammatory mediators and of each AA metabolite (LTB4, PGE2, TXB2 and 6-K-PGF1A) between samples derived from non-CF and CF subjects.


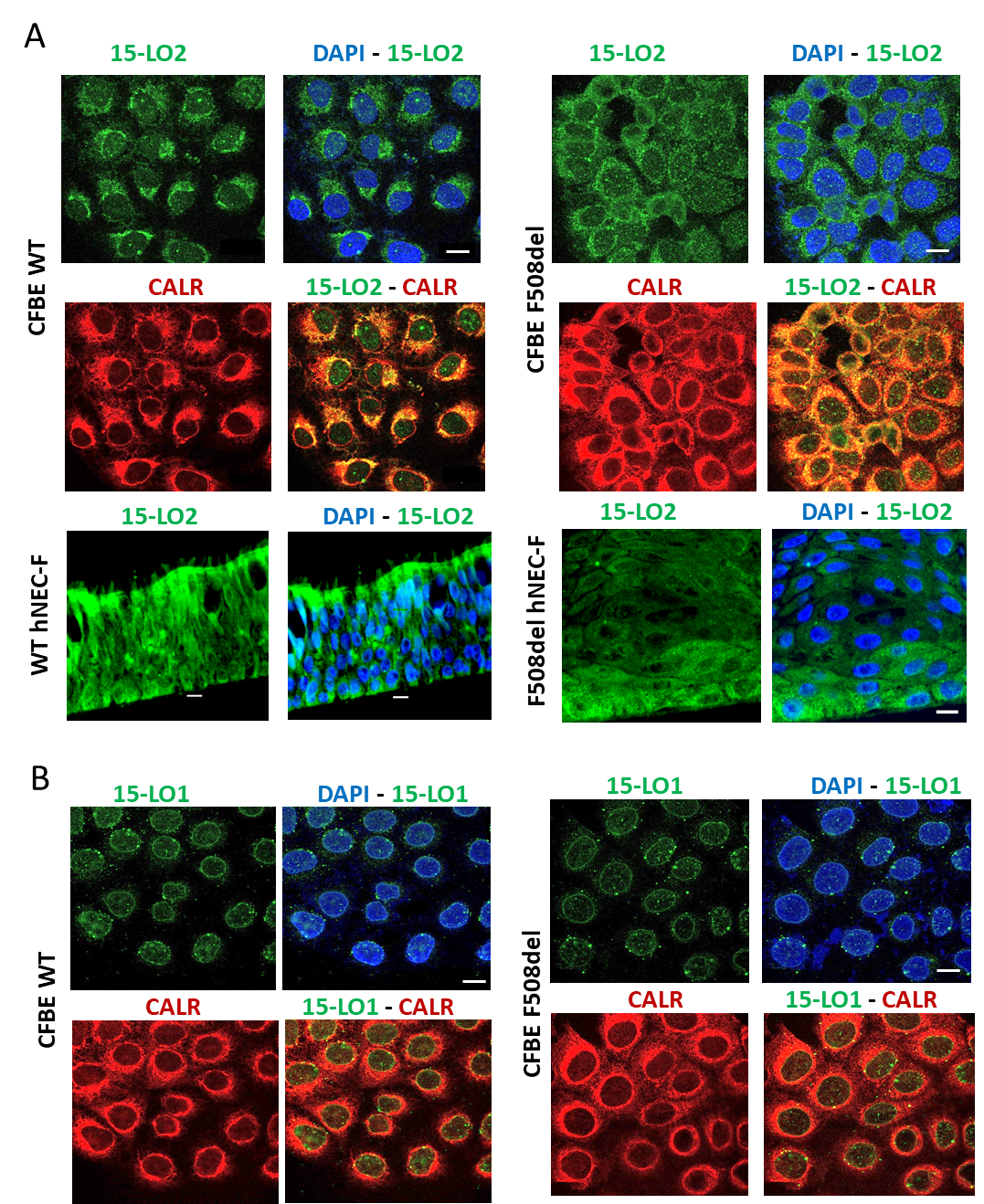


**Supplemental Figure 4.** 15-LO localization in CFBE41O- cell line and hNEC**. A)** Typical 15-LO2 (green) immuno-fluorescence confocal images of CFBE41O- cell line (grown on glass) and hNEC primary cultures (grown at ALI) expressing either WT CFTR (left) or F508del mutated CFTR (right)**. B)** Co-immunostaining of calreticulin (CALR, red) Typical 15-LO1 (green) immuno-fluorescence confocal images of CFBE41O- cell line (grown on glass) expressing either WT CFTR (left) or F508del mutated CFTR (right)**.** Nucleus were stained in DAPI (blue). Bar=10µm.

# Supplementary Table legend (table submitted as an excel file)

**Supplementary Table.**  Correlation analysis between lipids and enzymes expression level within the same hNEC culture.
